# Experiences of recovery and a new care pathway for people with pain after total knee replacement: qualitative research embedded in the STAR trial

**DOI:** 10.1186/s12891-022-05423-5

**Published:** 2022-05-13

**Authors:** Andrew Moore, Vikki Wylde, Julie Bruce, Nicholas Howells, Wendy Bertram, Christopher Eccleston, Rachael Gooberman-Hill

**Affiliations:** 1 Bristol Medical School, University of Bristol, Bristol, UK; 2 National Institute for Health Research Bristol Biomedical Research Centre, University Hospitals Bristol and Weston NHS Foundation Trust and the University of Bristol, Bristol, UK; 3 Warwick Clinical Trials Unit, Division of Health Sciences, University of Warwick, Coventry, UK; 4 North Bristol NHS Trust, Bristol, UK; 5 Centre for Pain Research, The University of Bath, Bath, UK; 6 Department of Clinical and Health Psychology, The University of Ghent, Ghent, Belgium

## Abstract

**Background:**

Approximately 20% of people experience chronic postsurgical pain after total knee replacement. The STAR randomised controlled trial (ISCRTN92545361) evaluated the clinical- and cost-effectiveness of a new multifaceted and personalised care pathway, compared with usual care, for people with pain at three months after total knee replacement. We report trial participants’ experiences of postoperative pain and the acceptability of the STAR care pathway, which consisted of an assessment clinic at three months, and up to six follow-up telephone calls over 12 months.

**Methods:**

Semi-structured interviews were conducted with 27 people (10 men, 17 women) between February 2018 and January 2020. Participants were sampled purposively from the care pathway intervention group and interviewed after completion of the final postoperative trial questionnaire at approximately 15 months after knee replacement. Interviews were audio-recorded, transcribed, anonymised and analysed using inductive thematic analysis.

**Findings:**

Many participants were unprepared for the severity and impact of postoperative pain, which they described as extreme and constant and that tested their physical and mental endurance. Participants identified ‘low points’ during their recovery, triggered by stiffening, pain or swelling that caused feelings of anxiety, depression, and pain catastrophising. Participants described the STAR assessment clinic as something that seemed “perfectly normal” suggesting it was seamlessly integrated into NHS care. Even in the context of some ongoing pain, the STAR care pathway had provided a source of support and an opportunity to discuss concerns about their ongoing recovery.

**Conclusions:**

People who have knee replacement may be unprepared for the severity and impact of postoperative pain, and the hard work of recovery afterwards. This highlights the challenges of preparing patients for total knee replacement and suggests that clinical attention is needed if exercise and mobilising is painful beyond the three month postoperative period. The STAR care pathway is acceptable to people with pain after total knee replacement.

**Supplementary Information:**

The online version contains supplementary material available at 10.1186/s12891-022-05423-5.

## Introduction

Total knee replacement is a common elective surgical procedure most often performed to improve mobility and relieve pain associated with osteoarthritis. Over 100,000 primary knee replacements are usually performed in the United Kingdom’s National Health Service (NHS) annually [1–3]. Although knee replacement reduces pain severity for many people, about 20% of those having this operation experience chronic pain afterwards [4, 5]. Chronic postsurgical pain is defined as pain developing or increasing in intensity at three months or longer after surgery [6]. People with chronic postsurgical pain after knee replacement are often dissatisfied with their outcome [3, 7], and struggle physically and mentally with their ongoing pain [8]. Some people describe that that they feel abandoned by healthcare services/professionals [9], or that the advice they receive is unhelpful if told that their pain is a normal part of recovery or that radiography shows no technical or mechanical reason for pain [10]. People who do not seek healthcare for chronic postsurgical knee pain may not do so because a complex mix of physical, social and psychological factors leads them to believe that nothing more can be done for their pain [10].

Current service provision for chronic pain after knee replacement is varied and inconsistent [11]. Complexities and variation in assessment and management of people with chronic postsurgical pain, coupled with a lack of explicit access points to services, means that clinicians often struggle to help [12]. There is an acknowledged need for a comprehensive multidisciplinary approach to manage chronic pain after knee replacement [13].

Based on this pressing need for individualised, targeted care, an applied research programme funded by the National Institute for Health Research (RP-PG-0613–20,001) developed a new care pathway that was evaluated in a pragmatic randomised controlled trial: the Support and Treatment After joint Replacement (STAR) trial [14].

The STAR trial, conducted in eight UK NHS hospitals, evaluated the clinical and cost-effectiveness of the new care pathway [15]. The pathway consisted of early postoperative screening to identify people with ongoing pain and an assessment clinic at three months postoperatively with a trained Extended Scope Practitioner (an allied healthcare professional with specialist orthopaedic training), with up to six follow-up telephone calls over 12 months as required. The 1-h assessment was held 3–4 months after the surgery and involved: clinical history taking, a review of patient reported outcomes including pain, depression, and neuropathic pain. A knee examination involved evaluating the sites and nature of knee tenderness, wound healing, signs of infection, range of motion, and signs of and symptoms of Complex Regional Pain Syndrome as per the Budapest Criteria [16]. Knee radiographs were taken to check implant fixation, alignment, evidence of fracture of concerns with sizing. Blood tests were taken for markers of infection. Further details of the pathway are provided in the previously published trial protocol [15]. The pathway enabled appropriate onwards referral to existing services which included any of the following: a surgeon, if pain was thought to attributable to surgical factors or infection; physiotherapy for exercise and mobility advice and support; a GP for further assessment of depression or anxiety; and/or pain specialists for neuropathic pain or Complex Regional Pain Syndrome (via GPs). Referrals aimed to ensure that underlying reasons for chronic pain were identified early, to prevent pain persistence and ensure that treatment is targeted at likely reasons for pain. The development and refinement of the new pathway and its potential for implementation is described elsewhere [4, 11, 12, 14].

The trial evaluated whether supplementing usual care with the new STAR care pathway improved pain outcomes for people with troublesome pain three months after total knee replacement when compared with usual care alone. The co-primary outcomes for the trial were pain severity and pain interference, assessed using the Brief Pain Inventory [17] at 12 months after randomisation, which was approximately 15 months after surgery. A total of 363 patients with troublesome pain at three months after surgery (defined as a score of ≤ 14 on the Oxford Knee Score Pain subscale [18]) were randomised; 242 to receive usual care and the STAR care pathway and 121 to receive usual care alone. Those who received the STAR care pathway had better pain outcomes than people who received usual care alone, and the care pathway was cost-effective [19].

Qualitative methods embedded within clinical trials can provide insight into how people view interventions, which can add another layer of insight to trial findings [20, 21]. In this article we describe findings from a qualitative study embedded in the STAR trial. The study aimed to explore trial participants’ experiences of the first 15 months after total knee replacement surgery and the STAR care pathway.

## Methods

One-to-one, semi-structured interviews were conducted between February 2018 and January 2020 with trial participants allocated to receive the STAR care pathway. A sample of 27 participants (10 men, 17 women) selected from the intervention group were interviewed after they had completed the final postoperative trial questionnaire, at 15 months after their surgery. A purposive sample of 30 participants was planned as an approximation expected to achieve data saturation, so that no new data were identified by the time that data collection was complete. This is in keeping with standards of qualitative research [22]. The sample was chosen purposively to include patients with a range of outcomes (pain severity and interference) to ensure a diversity of experience within the sample. The study received ethical approval from the South West – Central Bristol Research Ethics Committee in July 2016 (REC reference 16/SW/0154) and Health Research Authority approval in August 2016.

### Recruitment

Participants were sampled purposively to include people selected from six study sites, all NHS hospitals, across the North, Central and South of England and Wales, and to reflect diversity in outcomes for pain and interference over time, assessed through the Brief Pain Inventory. After return of the 15-month postoperative trial questionnaire, selected trial participants in the intervention group were then sent a letter of invitation and information about the qualitative study, after which they were contacted by telephone to discuss the study and arrange an interview.

### Data collection

All interviews were conducted face-to-face in participants’ homes between February 2018 and January 2020. Interviews were conducted by the lead author AJM (male, PhD) with a background in health sociology and extensive qualitative research experience. Interviews lasted between 24 and 76 min (mean: 45 min). All participants provided written informed consent before interview, including consent to audio-recording and publication of anonymised quotations. The topic guide was developed in collaboration with patient representatives (See Additional file 1) and covered experience of the postsurgical period, pain after surgery, experience of the STAR care pathway, and what participants judged had worked or not worked to improve their pain. The interviewer was able to follow up and ask further questions of participants if new or unexpected findings emerged. Interviews took place after participants had completed their final follow-up for the trial to reduce any risk that reflecting on their experiences might affect completion of their 15-month postoperative trial questionnaire.

### Analysis

Audio-recordings of the interviews were transcribed, anonymised and uploaded to NVivo 12 data management software [23]. Inductive thematic analysis was undertaken [24]. Transcripts were initially coded inductively on a line-by-line basis by AJM. Coding was descriptive in nature. As similarities and relationships between the codes were established, themes were developed. As coding became more conceptual, moving towards higher levels of abstraction, the themes were refined. For example, some participants described how in the postoperative phase they progressed until their knee began to stiffen or a different kind of pain began to develop, and they began to struggle physically and psychologically. As more participants began to describe similar experiences, this developed into the theme referred to as ‘Low points’. To ensure rigour in the coding process, four transcripts were independently coded and codes were agreed and applied. Double coding was by two other team members with different disciplinary perspectives: RGH (Professor of Health and Anthropology) and WB (Trial Manager).

## Results

### Participant characteristics

Twenty-seven participants were interviewed, from six of the eight trial sites. Two sites opened to recruitment in the latter part of the trial and their participants had not yet completed 15-month postoperative trial questionnaires by the time of the qualitative study. Out of 30 people invited, three did not take part: one decided that other health problems were their priority, and two did not respond to telephone calls. The final sample included 10 men and 17 women, aged 55 to 84 years (mean: 71 years) (Table 1). Change in pain scores varied across the sample (Table 2).

**Table 1:** Sample demographic characteristics

Age group, years	Number (n)
55–64	4 (15%)
65–74	15 (55%)
75–84	8 (30%)
Sex	
Male	10 (37%)
Female	17 (63%)
Hospital Site	
Site A	5 (18.5%)
Site B	4 (14.8%)
Site C	5(18.5%)
Site D	5 (18.5%)
Site E	4 (14.8%)
Site F	4 (14.8%)

**Table 2:** Individual participant characteristics

Pseudonym	Age range at interview (years)	BPI-S Score, baseline to 12-months^a^	BPI-I Score, baseline to 12-months^a^	Change in pain scores^b^
Helen	65–74	2.3–1.0	6.6–1.0	Improved
Tom	65–74	3.5–1.0	2.7–0.9	Improved
Chloe	55–64	3.5–0.5	4.3–0.1	Improved
Reg	75–84	4.0–3.0	5.7–1.6	Improved
Grace	65–74	4.5–0.5	4.3–0.0	Improved
John	65–74	4.8–0.2	5.0–0.6	Improved
Mandy	55–64	4.8–3.0	8.1–5.1	Improved
Clive	65–74	4.8–3.0	4.9–2.1	Improved
Catherine	75–84	5.0–0.0	6.4–1.1	Improved
Joan	75–84	5.0–3.5	9.4–7.7	Improved
Bernice	65–74	5.3–0.8	4.9–0.1	Improved
Deborah	65–74	5.5–2.0	7.1–4.6	Improved
Caroline	55–64	5.5–2.3	4.1–1.9	Improved
Bob	65–74	6.5–1.0	6.0–0.1	Improved
Rose	65–74	6.8–1.0	7.7–1.3	Improved
Andy	65–74	7.3–4.8	7.9–3.9	Improved
Anne	75–84	8.3–4.0	8.3–5.4	Improved
Elizabeth	65–74	8.5–3.0	7.0–2.7	Improved
Josie	65–74	7.0–6.0	7.6–7.7	Improved for severity; same for interference
Keith	75–84	5.8–5.0	6.3–1.0	Same for severity; improved for interference
Betty	65–74	5.8–5.0	6.0–3.4	Same for severity; improved for interference
Gary	75–84	4.8–4.3	4.3–4.3	No change
Graham	65–74	6.3–6.5	7.4–8.0	No change
Bonnie	65–74	7.8–7.0	9.6–8.6	No change
Louise	55–64	10.0–10.0	10.0–9.9	No change
Nora	75–84	3.3–5.8	8.0–9.0	Worse
Archie	75–84	3.5–5.2	7.7–8.9	Worse

^a^Pain scores range from 0–10 (best to worst)

^b^Based on minimally clinically important difference of 1 point on the Brief Pain Inventory Pain Severity (BPI-S) and Pain Interference (BPI-I); Worse = increase from baseline to 12 months of ≥ 1; No change = change in score from baseline to 12 months of < 1; Improved = decrease from baseline to 12 months of ≥ 1

### Main findings

Two thematic domains were developed: The first domain, entitled “The postsurgical journey” includes the severity of postoperative pain; endurance and the hard work of progress; and low points. The second domain describes the “Experiences of the STAR care pathway”, including the acceptability and the perceived benefits of the STAR care pathway. Illustrative quotations relating to our interpretation of experiences can be found in Table 3. All names are fictional to preserve anonymity.

**Table 3:** Illustrative quotations

**Theme & subthemes**	**Illustrative Quotations with Pseudonyms**
**The postsurgical journey**	
Severity of postoperative pain	“The pain is horrendous. It is horrendous, you know, and I was, like I said, on my own. I mean I had friends coming in for the first few days and afterwards but you're sat on your own some days and you—I mean I, I think I've screamed about twice in my life […] but yeah, I mean the pain is—yeah, it's extremely painful” (Clive) “I remember saying to [surgeon] when I last saw him and I said I’d rather have ten babies than ever have to go through this again, and he said you are not the first one to say that […] it’s so painful, it’s horrendous and it’s constant.” (Grace) “I knew it was painful but I thought oh well for me I’ve got a strong pain threshold, but this has beat me ain’t it just […] it’s worse than childbirth because childbirth is over in a matter of you know minutes sort of thing whereas this goes on and on and on.” (Josie) “It’s a most terrible pain that you aren’t prepared for in any way […] [*was it sort of hours after the surgery or days, or weeks?*] Months, mine went on until, well I suppose it started improving and subsiding a bit after about six months […] we had morphine [in hospital], although morphine didn’t seem to do anything, I was amazed how little effect any pain relief had, but I was able to do things, you know all the things that the physiotherapist said to do, even though it was incredibly painful.” (Deborah) “I forgot it was as bad as this! [laughing] Then they bring you the crutches and they say right, come on you’ve got to walk a few steps now like, me and the girl [in] the bed across the road and she went, I forgot about this, did you?” (Chloe) “Once it’s all gone you tend to forget the pain don’t you.” (John) “I put there, 'Much more painful than I ever anticipated.' So that was the first couple of weeks […] [*It sounds like the first bit was quite tough*] It was, yes. It was. Because I've actually broken lots of bones over the years … I've had three children, I thought that my pain threshold was quite high […] [*Does this top it all?*] Well certainly it did, yes […] Because it actually is very hard. If it wasn't for these notes I wouldn't remember.” (Helen) “I suppose thinking right back to the beginning I think more understanding of how it might be, you know. I think if I'd been given more information about the possible outcomes, how painful it might be.” (Helen)
Endurance and the hard work of progress	“My knee is absolutely great. Really happy with it. I need the other knee done but it will have to be hanging off before I have it done. I think it needs to be really bad for you to appreciate the pain that you go through to get it better […] I would tell anybody, ‘Please have it done. It improves your life so much more.’ So definitely. As long as you’re willing to put in the work to – to do that." (Caroline) “Had the pain been a lower level I could have managed it, but I couldn’t manage that level because for the whole time up to, for at least six months, I wasn’t able to sleep. I had to get up several times during the night and come down for ice packs and God knows what and, sort of move about. If I was still at all it just took over, so I had to move and do anything I could to distract myself and alleviate it.” (Deborah) “The pain when you’re trying to bend it makes you cry but you’ve got to keep doing it and I don’t think [friend] did that you see and now she walks with a stick.” (Chloe) “Yeah the physios were great but it was painful when I went there, I used to dread it because I knew it was going to hurt because they start saying ‘oh you need to do this and you need to do that’, and I wasn’t always good at doing exercises because it was going to hurt.” (Mandy) “I got frustrated at times but then, you know, you've got to sit back and think, 'Well, this is part of the process and that's the end of it.'” (Clive) "Probably reached the plateau. Yeah, the first six months was the hardest, that was for sure. But it’s settled down now, yeah." (Reg) "I’ve been doing all the exercises that I was supposed to be doing, but I started going out and walking twice a day and it was just a complete turnaround. I think mentally as well. I felt I was really taking back ownership of my knee […] I felt, ‘Okay, the surgeon has done his bit and its sort of like his knee. He’s put the part in. He’s done this bit,’ but I suppose it’s – it’s also sort of like taking back part of my life […] So, I think because I had decided, ‘this is what I’m gonna do,’ that is where I felt like I was taking back ownership a bit more. ‘It’s my knee. I need to sort it.’" (Caroline)
Low points	"I think if – if you’re gonna put the time in, but I did say, week 3, I felt like a tonne of bricks ‘cause everything just stiffened right up […] That was my real low point, where I thought, ‘Oh god. Is this it?’ but luckily enough that didn't last sort of too long, but that – that was a very low point, I have to say.” (Caroline) "It wasn't the pain that got worse. It was the stiffness, which then was a sort of pain, but it wasn't the sort of pain of when you’ve just had the operation. As I say, it was only week 3, but to me that was quite a way on, it felt like at that time […] but yeah, it was the actual stiffness and the mobility side, I felt like suddenly I was going backwards [Was that what worried you?] That really worried me. I thought, ‘Why am I – why is this happening? I’m doing my exercises. This shouldn't happen.'" (Caroline) “I've put here, 'The knee bend seems to be completely stuck at 90 degrees at four weeks. I feel weak, hopeless and depressed, not seeming to make any progress.' So, I think for those next few weeks it was tough […] So, four weeks I was really fed up, five weeks I'd started saying, 'Still little or no progress.' Six weeks a bit of improvement. I think at ten weeks I was telling my physio that I, I couldn't—I thought there was something really wrong with it.” (Helen) “For the first four or five weeks, I really felt I was making progress. I was full of optimism. I was walking around the garden with very little pain really […] I had plenty of movement in it and I was thrilled to bits. We went out for the day […] We didn't overdo it, but that was the first time it blew up like a balloon and got all hot and red, and I told them about that at [STAR clinic] and I mean they showed me the X-ray and they said, 'The X-rays looks—it's all in perfect position.” (Nora) “Yes because when you’re doing it at first they give you exercises […] and like they say you’re doing it so far […] but to make it go far enough you’ve got to push it that bit further and that bit further and that bit further all the time and it gets to a point where it stiffens up and you don’t want to do it, and I think you’ve just got to get by that barrier because then it gets easier, easier to do.” (Chloe)
**Experience of the STAR care pathway**	
Acceptability of the STAR assessment clinic	“I’m not sure, there were occasions where I wasn’t sure where the clinic stopped and the other one started.” (Bernice) “It just seemed perfectly normal, really, that, you know, you'd have x-rays and they said they were okay, and physio said it was okay and so it was—yeah.” (Helen) "I was told that I might get extra help with the STAR Clinic but I'm not really sure what extra help I've had through, through the STAR Clinic really […] I mean whether because I was taking part in it… I had the referral to the Pain Clinic or whether I would have the referral to the Pain Clinic anyway, I don't know." (Nora) “Loads of questions, saw a physiotherapist, they took blood […] I had loads of x-rays and someone wasn’t happy with some of the x-rays, so I had to go back down and have more x-rays. They were looking at it proper like you know […] it was a thorough examination. And very pleasant, nice cuppa tea, being fussed over, yeah I mean I must have done an hour’s interview with the physiotherapist because she recommended the physiotherapy.” (Graham) “I was really quite impressed with it and I actually found it really useful because I felt as if I’d got somebody to go and ask questions to … it was really nice because I had a bit of an MOT in a way and … I just thought I’ve actually had a bit of a check-up but it was really good to be able to talk to her about you know things about my knee that I was concerned about … I know I was there quite a long time with her and they called me back for another blood test because there was a bit of infection or inflammation which I mean it was still inflamed anyway so that was… but I just felt as if I was well looked after so…” (Mandy) "I was made really welcome […] So, erm, I had a cup of coffee and taken down, I had a cup of chocolate. Straight in and, you know, no waiting in the waiting room, all this. Really nice, and I tried to answer as many questions as I can and, which I did and there was a lot of questions, you know. Quite a few questions. No, very good and, er, told me they’d see me again and such, or you, I’d be hearing from them you know. Kept all the information in me folder, all the feedback and everything […] I had, yes, blood tests done. I had an X-ray done and they showed me them all afterwards and everything with it because they knew I was a nurse, you know, and they showed me the X-rays." (Elizabeth) "It seemed to be run very efficiently and everything, so, erm, you know, I was quite happy. I didn't find it overly intrusive time-wise, or anything like that, ‘cause that was one of my worries, when I was going to the hospital first of all I thought I had to go to the hospital several times and they said, ‘No, no, no.’" (Caroline)
Perceived benefits of the STAR assessment clinic and follow-up calls	“It’s sort of gave me encouragement to sort of well, these other people are looking after me as well and treating me. They are kind and I felt there was a, I needed to get myself right and do what little things they suggested, you know, and then, you know, you, mentally you, I felt more possible.” (Elizabeth) “[It was] just like talking really, mainly, just talking. Put me mind at rest with things as well […] Because I was in a lot of pain at first, but they did help with talking, when they were talking to me and they could let me ask questions as well, which was good, yes.” (Anne) “They were spot on. They asked some good questions, you know and they listened which you know – so.” (Andy) “[*the follow-up phone calls that you had, how did you find those?*] I mean it was fine; it was someone checking up on me really. You know, from an orthopaedic clinic you don’t really get anything […] I mean they were very good because when I first went they did arrange an x-ray, they did arrange physio and beyond that I didn’t really need anything else. And they’ve rung me up several times to see how I am, I can’t fault it really.” (Rose) “I would have gone to the hospital you know, thinking there was really something wrong, but he just tried to reassure me and you know and they’ve all put it down to muscle… muscular nerve pain, that’s what I believe, the impression I seemed to get so… […] I say that was the first and only time I’ve ever rang [ESP] apart from him just ringing me and checking me, checking how I was you know which was very nice because I didn’t expect that to happen at all.” (Grace) “It’s not attached properly [the knee prosthesis] so it’s slightly different from other people who are just experiencing continuous pain for no obvious reason and I feel fortunate in one way that I know what’s the matter because you do need to know what’s the matter don’t you otherwise you think you are going mad in the end […] there was a suggestion of having another replacement, I just couldn’t face it so I’m happy to go… you know I can manage this, it’s painful but I’d prefer to manage this because it is manageable pain.” (Deborah) “I think we’re finding out about this alignment. Yeah and I thought it was just me you know, not taking to the knee properly and it could’ve been that not doing its job. Yeah, I thought they come over spot on, you know what I mean? It were very good and I’d do it again.” (Andy) “Probably gave me confidence that it weren’t me because you think like the information you think it’s your fault because you’re not doing something right you know what I mean? Although you’re doing your exercises you have no yard stick to measure anything to so you don’t know so when I went to see him and he said well it’s brilliant, your leg is brilliant, carry on with what you’re doing.” (Chloe)

### The postsurgical journey

#### Severity of postoperative pain

The majority of participants spoke about the intensity of postoperative pain, describing it as extreme and constant. A common feature of participants’ accounts was the unremitting and constant nature of pain in the first few weeks postoperatively, up to three months and beyond. Without a reference point such as previous surgery, it is understandable that patients might underestimate the likely degree of pain, but even those who had previous knee replacement surgery struggled to remember how painful it was. Helen kept a diary during her recovery and reflected that she would have forgotten how intense the pain was if she had not documented it. A number of participants believed themselves to have high pain thresholds but were surprised by the intensity and duration of the pain, Josie describing herself as “beaten” by the pain. Participants suggested they would have liked to have had more information before the surgery about pain intensity afterwards.

#### Endurance and the hard work of progress

When describing their postoperative recovery, participants often spoke about the physical and mental effort required to endure their pain. Caroline described how her experience of recovery had tested her endurance, and that she would recommend surgery only for those “willing to put in the work”. Mandy and Chloe cautioned that unless one could endure and push through the pain, the final outcome could be worse.

Some participants endured pain for long periods, which led to problems sleeping. Deborah described enduring intense postoperative pain for six months, which had affected her sleep as she had to apply ice packs several times throughout the night and keep herself moving to distract herself from the pain. Clive described frustration at his lack of progress, before reasoning and accepting that it was “a part of the process”. Enduring pain for prolonged periods was described as an essential element of recovery.

Caroline described a division of labour which was related to taking back ownership of ‘the knee’. She saw the knee as belonging to the surgeon, initially—“it’s sort of like his knee. He’s put the part in”—but then having worked through her prescribed exercises, she started to do her own exercises. She described feeling a need to take control of her own recovery, and in doing so taking back ownership of her knee, which seemed to be physically and psychologically important to her progression, and part of the ‘work’ of a positive recovery.

#### Low Points

Many participants described how during the first three months after surgery, and sometimes beyond, they had experienced negative emotions, or a “low point”, typically periods of low mood, feeling depressed and anxious, or worried about their progress. They described a number of reasons for low mood, including: pain; sudden onset of stiffness or swelling; and the realisation that they were not progressing as well as they had hoped, which led to confusion and uncertainty about ongoing symptoms.

The sudden onset of knee stiffening during her third week, led Caroline to feel that she was “going backwards” rather than recovering. This worried her and she felt uncertain about why this apparent regression was happening, despite her adherence to prescribed exercises. Helen also described feeling “weak, hopeless and depressed” at her lack of progress, and by the tenth postoperative week had wondered if something was “really wrong” with her knee. However, by the twelfth week she had begun to make progress in her recovery.

Nora described how she initially felt full of optimism, making good progress in the first five weeks, before then experiencing sudden swelling, which derailed her progression. Chloe also experienced low points related to the onset of stiffening and how this made her exercises difficult and more painful. She described the need to push past this barrier to progress.

### Experiences of the STAR care pathway

#### Acceptability of the STAR assessment clinic and follow-up calls

Impressions of the STAR postoperative assessment clinic varied. Some participants remembered the clinic’s comprehensive and “thorough” approach, characterised by the amount of questions they were asked and radiographs taken. Participants also appreciated the opportunity to ask questions and to talk about their concerns at length with a specialist clinician. Mandy suggested this gave her a sense of being “well looked after”. Some participants spoke of being made to feel welcome, and having a sense of being “fussed over”. Despite the clinic assessment taking an hour or so, Caroline reflected on how efficiently it was run and that it was “not overly intrusive time-wise.”

Other participants saw the STAR assessment clinic as “perfectly normal” while some struggled to remember the clinic as it did not stand out as different to them, from other care they had received. Others were not sure whether they had benefitted specifically from the clinic review, partly because they were unsure which elements of the clinic were novel and how these differed from usual care. For example, Nora wondered if she would have received a referral to the pain clinic regardless of whether she had attended the STAR clinic, and so was unsure whether the clinic had helped. Nora was referred to pain management services after assessment by the STAR clinic. She also received six follow-up telephone calls from the STAR Extended Scope Practitioner, to review her progress and give further advice on pacing activity. It seems likely that because most participants had no previous experience of knee replacement, they saw the STAR clinic as seamlessly integrated into the care that they would usually expect to receive from the NHS. This contributed to the acceptability of the STAR clinic and subsequent follow-up.

#### Perceived benefits of the STAR assessment clinic and follow-up

We explored which elements of the STAR care pathway worked best for participants. Participants described how the assessment clinic referred them to other treatments/services, but also benefited from psychological support through the efforts of healthcare professionals taking time to focus on their pain and recovery, and to provide care during this assessment.

Elizabeth described feeling encouraged in her efforts to recover because she felt “looked after” by the STAR clinic team and she expressed motivation to reciprocate the efforts of the team by “getting herself right”. Similarly, Anne and Grace described how talking with the STAR Extended Scope Practitioner and the follow-up calls put their minds at rest about their pain, with Grace suggesting that had she not spoken to the ESP she would have gone to hospital otherwise. Others also reported being actively listened to and were reassured by being “checked up on” by clinic staff.

Some participants described how even though subsequent treatments had not resolved their pain, they felt reassured because of the information they had received about the possible cause of their pain. It appears this sense of certainty made their pain more acceptable. For example, Deborah was referred to her orthopaedic surgeon for suspected malalignment who discovered that the prosthesis had indeed worked loose. She described feeling “fortunate” to know the reason for her pain, rather than “going mad” with uncertainty. Similarly, Andy was referred to his surgeon for suspected malalignment. To Andy, knowing the reason for his ongoing pain and that he was not to blame, was a benefit of the STAR clinic. Chloe also derived confidence from the STAR clinic, as it helped her to understand that the pain was not her fault, and that she should continue with her exercises. Although designed to assess and attend to individuals’ needs and to tailor support to reasons for pain, the STAR pathway had not explicitly set out to provide psychological support. This finding was unexpected yet a common experience.

## Discussion

We explored the experience of postoperative recovery and of a new care pathway for chronic pain after knee replacement. Previous research shows that patients often struggle with pain in the postoperative period, and that support for ongoing pain following surgery is lacking [9, 10]. Although some participants described ongoing pain, they nonetheless found the STAR care pathway acceptable and provided them with reassurance and confidence in their ongoing recovery. Participants’ descriptions of the STAR clinic as something they would expect to receive, and “perfectly normal” suggests the clinic was seamlessly integrated into NHS care and normalised, at least from the perspective of people with pain. This holds promise for the future implementation potential of the STAR care pathway. Healthcare professionals’ perspectives on the implementation of the STAR care pathway will be reported separately.

Participants valued the opportunity to discuss concerns with a health professional and derived reassurance and encouragement from the clinic and follow-up telephone calls. Self-blame has been acknowledged as a prominent rationalisation for ongoing pain amongst people who have received a total knee replacement [8, 9], and we found that some patients, having received an explanation for their pain during the STAR clinic, felt reassured that it was not their fault. The timeliness of this support and education is important as other recent studies show that support within the three month post-surgery period, could improve patients’ chances of better long-term outcomes [25, 26].

A key finding of the study is that many patients were unprepared for the severity and impact of acute postoperative pain, and this seemed to characterise the recovery of those with suspected chronic postsurgical pain. This aligns with evidence that severe acute postoperative pain is a predictor of chronic postsurgical pain [27–29] and whilst many patients recover as expected, a sub-group who experience severe postoperative pain may be most likely to develop chronic postsurgical pain.

While some patients suggested they would have liked more information preoperatively about how much pain they might experience, even those who had previous knee replacement surgery had forgotten the intensity of acute postoperative pain. The phenomenon of diminishing pain recall has been reported in other types of surgery and in experiences of childbirth [30–32]. Our findings further highlight the challenges of preparing people for the intensity and duration of pain they may experience after total knee replacement. In the NHS, patients often receive information during a preoperative clinic about what to expect before and after surgery. Future research might focus on understanding and improving information delivery at preoperative clinics.

Another key finding was our identification of a period between the acute postoperative and three-month time points, during which participants experienced a low point, characterised by depression and anxiety associated with a plateauing of progress, and onset of stiffness, swelling or pain. Standard six-week follow-up appointments often focus on radiological evidence, and if there is no mechanical explanation for ongoing pain, patients can feel uncertain about their progress and fearful about the future [10]. Rehabilitative support during this early postoperative period can be variable [33] and continuity of support is often absent. Participants in our study described pushing through this low point, and different aspects of the experience of postoperative pain related to the idea of recovery as ‘hard work’, requiring both physical effort and the ability to endure and tolerate pain. This might suggest that preventive interventions for pain are required beyond the early postoperative period if exercise and mobilising is painful. The concept of physical and emotional ‘hard work’ has previously been identified in studies of self-management of painful osteoarthritis [34]. We find that the concept is also relevant to the postoperative experience and that the STAR assessment clinic was an opportunity to provide reassurance and encouragement to people who experience these low points and who may have concerns about their progress. May and colleagues suggest that patients have an increasingly important role in managing their illness and recovery “the new proactive work of patient-hood” [35]; the STAR care pathway may enable patients to play a greater part in the management of their recovery. Participants’ account of their postoperative experience and the STAR care pathway suggests that rather than being thought of as a one-off event done *to* the patient, total knee replacement may be more usefully seen as a treatment that lasts beyond the surgical procedure, requiring multiple specialist input *and* patient self-management, until patients return as near as possible to their desired state of health. Our findings show the STAR care pathway can ensure the provision of multiple specialist input and provide people with confidence to manage their own recovery.

### Strengths and limitations

This study fulfils the criteria specified in the Standards for Reporting Qualitative Research (SRQR), a 21-item checklist for qualitative research [36]. A qualitative approach enabled us to explore the complexity of people’s varied experiences of the STAR care pathway. Our inductive approach allowed us to address the study aims and to identify areas of relevance to study participants. The sample included 27 participants from six hospitals. Participants had diverse outcomes for pain intensity and interference over time, and all had pain after their knee replacement. The sample size provided sufficient data to achieve data saturation, the point at which the collection of further data was unnecessary [22]. The demographics of the trial sample in terms of ethnicity (100% white), mean age (71 years) and gender (63% female) broadly reflects the national population of individuals undergoing total knee replacement at the time of the study (95% white, mean age 69 years, 57% female) [37]. However, a weakness of the study is that the participants were all white, and understanding the experience and acceptability of the STAR care pathway from the perspective of people of other ethnicities would strengthen its validity. Interviewing participants more than 15 months post-surgery may introduce some recall bias. However, patients attended the STAR assessment clinic at 3 months post-surgery with up to 6 follow-up calls over 12 months, which will have reinforced their experiences over time. We are confident that we have identified the aspects most salient to experiences of the STAR intervention and that participants found it acceptable.

In conclusion, we found that after surgery people say that they were unprepared for the severity and impact of postoperative pain after knee replacement. This further highlights the challenges of preparing people for total knee replacement surgery and future research efforts should focus on the best ways of achieving this. We also identified a period between the acute postoperative and three-month time point during which many patients were pushed to the limits of their physical and mental endurance, suggesting that interventions for pain could be recommended beyond the acute postoperative period if pain continues to interfere with exercise and recovery. The STAR care pathway is an acceptable source of care and support for people with chronic postsurgical pain and can provide reassurance and confidence in their ongoing recovery while ensuring they that they receive the most appropriate management for their pain.

## Supplementary Information

**Additional file 1.** STAR Topic Guide: Participant experience of STAR.

## Data Availability

Anonymised data may be shared via the University of Bristol Research Data Repository (https://data.bris.ac.uk/data/). Access to the data will be restricted to ensure that data is only made available to bona fide researchers for ethically approved research projects, on the understanding that confidentiality will be maintained and after a Data Access Agreement has been signed by an institutional signatory.

## Abbreviations

ESP: Extended Scope Practitioner
NHS: National Health Service
STAR: Support and Treatment After knee Replacement
