# Supplementary material for: Experiences of recovery and a new care pathway for people with pain after total knee replacement: qualitative research embedded in the STAR trial
Source: BMC Musculoskelet Disord. 2022 May 13;23:451. doi: 10.1186/s12891-022-05423-5 (PMC9103301; doi:10.1186/s12891-022-05423-5)
Supplement: Supplementary file 1 — Additional file 1. STAR Topic Guide: Participant experience of STAR. [file 12891_2022_5423_MOESM1_ESM.pdf]

Additional file 1

STAR Topic Guide: Participant experience of STAR

Introduction: Discuss how the interview will be recorded, issues of confidentiality, anonymisation and the aim of the research. The aim of this interview is to find out what people thought of the care that they received after their knee replacement operation, and what worked best for them.

Do you have any questions at this point or are you happy to start?

Part 1: General experience

- First, please can you tell me about how your knee is now? (probes: pain severity, activity level, valued activities, impact on others)
- Thinking back in time, can you tell me about your experience of arthritis in your knee before your operation? (probes: pain, activity level, valued activities, impact on others)
- Can you tell me about any other health concerns or issues that you think I might need to know about?
- Please can you talk me through your knee operation and how you felt afterwards? (probes: from immediately after the operation, through the days in hospital and then at discharge)
- Did you have a 6 week follow up appointment, at 6 weeks after your operation? If so then what happened there? What happened next?

Part 2: Experience of the intervention

- You were invited to take part in the STAR project (trial) and to come to a clinic about your knee. What did you think of the clinic that you went to (probes: organisation, expectations, referrals that were made)
- What happened next? What did you think of the things that were offered to you?
- Of these things that you were offered, can you tell me what worked best for your knee? And what worked least well for you?
- What were you hoping for from the clinic?

Part 3: Reason for pain and other areas

- Thinking back over what has worked and not worked, what do you think was the reason for you having pain after your operation? At the time, what did you think the reason was?

Part 4: Thanks and close

- Are there other things that you would like to tell me?
- Thank you for taking part

END
